# Stammering as It Occurred in the War

**Published:** 1919-12

**Authors:** R. G. Gordon

**Affiliations:** Late Captain, R.A.M.C.


### STAMMERING AS IT OCCURRED IN THE WAR.

BY

R. G. Gordon, M.D., B.Sc., M.R.C.P. Ed.,

*Late Captain, R.A.M.C.*

---

During the war stammering was a common neurosis in soldiers. It was met with either as a fresh symptom which had never been manifested by the patient in earlier life, as an aggravation of a pre-existing condition, or as a relapse of a disability which had been in abeyance for many years.

The importance of stammering varied with the rank and duties of the soldier; less significant in the private, it was of great importance in the officer or non-commissioned officer.

Now that all ranks are returning to civil life, this disability is a trying one, for in searching for work a man naturally feels himself handicapped if he cannot freely express himself in speech.

In my experience stammering is seldom a mono-symptomatic neurosis, but is accompanied by a certain degree of psychasthenia, manifested by insomnia and night-mares. In this respect it differs from aphonia, the other common functional speech defect, which is not, as a rule, associated with any interference with the mental faculties. Stammering itself, however, is a true hysterical symptom in the sense of Babinski's[1] definition, that hysteria is a condition in which symptoms are present which have been produced by suggestion.

Roussy and L'Hermitte[2] attempt to distinguish between real stammering and hysterical stuttering, but this seems to be an artificial division of a symptom which has a common origin, however and whenever it has arisen.

In the discussion of any hysterical condition, it is necessary to consider what factor originated the suggestion that has been accepted by the patient, and what are the physical and mental abnormalities induced by that suggestion.

A stammer may be acquired in three ways, either under the stress of some emotion, or by imitation, or in the course of recovery from mutism or aphonia. The emotional disturbances which tend to induce stammering are fear and anger, and especially fear. At this point it may be as well to make it quite clear what one means by fear. The emotion of fear is not synonymous with cowardice. Almost everyone is frightened by a sudden loud noise or unexpected blow, but most people quickly control the fear and therefore their resultant action is not "panicky," and they are not cowards. Thus almost every soldier was constantly being frightened, but very few were cowards.

Many observers from Darwin to Cannon have increased our knowledge of the results of the emotions, and one of the principal expressions of the two most dynamic emotions is an increase in muscular tone. This is at first a beneficial preparation for the co-ordinated action of an attack or retreat, but if this co-ordinated action is prevented, then the energy which ought to be so used is diverted in "expressions" which become exaggerated and detrimental. Amongst other things the hypertonus of the muscles becomes an actual rigidity.

If this affects the muscles of respiration and voice production, stammering may temporarily ensue as a secondary expression of the emotion. Normally this will pass off when the individual regains control over his emotion; but if his suggestibility is high, or temporarily rendered high by confusion or other cause, he may accept the suggestion that stammering has come to stay, and will continue to suffer from this disability.

Imitation is an innate tendency, stronger in some individuals than in others, and more influential in children than in adults. Stammering is often acquired in childhood as the result of imitation, whether of an actual stammerer or of what Scripture[3] calls a "clutterer."

This term is applied to those who speak so rapidly that their words are slurred into each other. If a young child attempts to imitate this he will almost certainly stammer, for the attempt to speak so rapidly will result in spasm of the muscles of the voice production. I have seen several cases where such a history has been quite definitely obtained.

Stammering as a result of imitation also occurred amongst soldiers, just in the same way as there occurred hysterical gaits and tics as the result of copying others in the wards.

When a soldier has become hysterically mute or aphonic, he very naturally comes to the conclusion that there is some marked obstacle which prevents him talking. If then in the course of treatment as the result of accident he finds he can frame some words or phonate, he is still convinced that he cannot talk properly. So he continues to commit errors in voice production, the result of which is a stammer, which if allowed to develop may be remarkably intractable. Hence, when treating a mute or an aphonic, if he tends to stammer instead of talking properly after regaining his voice, he should never be left in this stage, but treatment should be continued till he talks normally.

Such is the pathogenesis of stammering.

The physical factors will be found to consist of rigidity and wrong use of the muscles of respiration of the larynx and of the mouth. As a rule the muscles in all these regions are at fault at one and the same time, though often one or other group predominates. If the chest muscles are spasmodic, the patient fails to get anything out at all for some instants, then the sentence comes with a rush, the words tumbling over each other in the attempt to complete them before the breath stops again. If the laryngeal and palatal muscles are chiefly at fault, there will be defects over initial vowels, and the harsh, monotonous tone in the voice which is characteristic of stammerers will be noticed. If the action of the labial muscles is spasmodic, stuttering over initial consonants will be the predominant feature of the condition.

The breathing of the stammerer is almost always deficient. If his lung power is tested on a pneumograph, of which the average individual can turn the scale to 240—340, the stammerer will only succeed in turning it to 100—200 ; moreover, he does not use what breath he has correctly. In voice production phonation should occur during expiration, but the stammerer either tries to produce his voice during inspiration or when the breath is held. Frequently, in addition to the spasms of the muscles involved in speech, there is a "tic" of the facial muscles, so that the patient makes the strangest grimaces when he tries to talk, and in severe cases the arms and even the whole body may share in the universal inco-ordination of muscular action.

Such are the physical symptoms of stammering ; but almost more important than this, because of its great influence on the course of treatment of the trouble, is the mental factor.

This consists in the development of the dread of stammering. The patient loses confidence in his ability to get the words out, so he tries to rush his words before breath fails him. This accounts for the fact that all stammerers talk too fast. This mental symptom is the one which is the most resistant to treatment, and many a "cured" stammerer feels this dread, but by means of some trick manages to avert disaster and prevent his infirmity being discoverable to a casual acquaintance.

One patient of mine, who had a very slight stammer amongst other neurotic symptoms, had a dread of certain words, but he was often able to see them coming in the sentence he was speaking, and having a somewhat agile mind, was able to substitute some other word and thus avert his stammer.

The prognosis of stammering depends on various factors. Those men who have acquired their stammer *de novo* in the war ought to be cured easily, and I have had some cases who have been completely cured within half an hour. Those who have relapsed during the war, *i.e.* those who have once stammered but have lived a part of their lives without stammering, should be relieved again, but their treatment may take longer owing to the greater intensity of the mental dread of stammering. Some of those who have always stammered can be cured, others relieved so that their speech is better than before the war, while all ought to be raised at least to their pre-war level. The ease of treatment will depend on the amount of intelligent co-operation on the part of the patient, and in severe cases on the amount of perseverance and practice he is prepared to devote to this cure.

In treating the stammerer the first necessity is to establish a bond of confidence between doctor and patient, and the latter must be assured that his condition is anything but hopeless. Next it is desirable to find out how the stammer originated, and explain to the patient the pathogenesis of his condition. This to my mind is an essential in the treatment of all neuroses, and while investigating the origin of his stammer one can inquire into and explain to him the nature of his insomnia and dreams, which often co-exist. The patient must next be instructed as to the faults which determine stammering, viz. too great rapidity of speech and the incorrect use of the breath. He should be placed in a recumbent position with his whole body relaxed, so that he may in this way counteract any tendency to spasm. He is then instructed to take long breaths, and his inefficiency in this respect may be demonstrated to him on a pneumograph, if such an instrument is handy. Once he acquires the art of deep breathing he is told that if he speaks during easy expiration he cannot stammer, and he is instructed to say the word " one " during a long expiration, the breath being emitted " through the word." He is told to count up to ten, taking a long breath between each word, and he goes on at this until he can do it perfectly. He then takes a long breath and says "one, two" in the single expiration, then "three, four," and so on up to ten. When this is perfect he must enunciate "one, two, three" in one breath, then "four, five, six," and so on up to twelve. Next, four numbers in a breath, then five, and so on till he can count from "one" to "ten" during a single expiration without hesitating. Then in exactly the same way he says his name and address, and short sentences of various kinds. He is next given something simple to read aloud, first in the presence of the doctor alone, and then if possible in the presence of others. In favourable cases this may all be done at one sitting; in less favourable circumstances it may take some time to reach this stage.

When the patient is reading the doctor must insist that he reads slowly, takes plenty of breaths, and never attempts to speak except during expiration. These breathing rules, besides counteracting the actual physical faults, divert the patient's attention from the possibility of stammering, and so relieve the mental factor, and often after cure is practically complete the patient can make use of these breathing rules to avert a stammer which he feels is coming.

In cases where the harsh, monotonous voice due to laryngeal spasm is a prominent feature Scripture's[3] "octave twist" is of great use. The modulation of ordinary speech varies approximately through the interval of a third. By exaggerating this interval to the octave the spasm is overcome. The patient does this by reading aloud, causing the note of each syllable to differ from the last by at least an octave—high, low, high, low, and so on. He should not sing this so much as say it; the distinction is expressed in French by the words "chanteuse" and "diseuse." It is remarkable how soon this exercise improves the expression and modulation of the voice.

Where initial consonant stutter is the trouble another trick is useful. Suppose the patient has a difficulty with "b," so that he says "b-b-b-butter," it will be found that he can say "utter" quite well. He is therefore instructed to put his lips together and say "utter." The result is (b)utter, and after a little practice he achieves "butter." The same can be done with any other consonant, by getting him to place his lips and tongue into the requisite position and then say the word without the first letter.

So far as the mental factor of the loss of confidence is concerned, the patient requires unending encouragement, every improvement must be demonstrated to him, and he must be assured of further success as he finds he can talk better. Thus he gains confidence, so that he is able to talk without any self-consciousness at all, or with so little that he can control it and prevent the stammer by some trick, which he holds in readiness. These methods have on the whole proved very satisfactory in war cases, and have often improved or cured a life-long stammerer, so that it would seem desirable that we should extend the lessons we have learnt during the war to help our civilian patients.

REFERENCES.

[1] J. Babinski and J. Froment, *Pithiatisme et troubles reflex nerveuses.* Collection Horizon. Paris, 1916.

[2] Roussy and L'Hermitte, *Psychonevroses de Guerre.* Collection Horizon. Paris, 1916.

[3] Scripture, *Stammering and Lisping.* New York, 1915.

